# Complete genome sequence of *Bacillus cereus* FORC_005, a food-borne pathogen from the soy sauce braised fish-cake with quail-egg

**DOI:** 10.1186/s40793-015-0094-x

**Published:** 2015-11-11

**Authors:** Dong-Hoon Lee, Hye Rim Kim, Han Young Chung, Jong Gyu Lim, Suyeon Kim, Se Keun Kim, Hye-Jin Ku, Heebal Kim, Sangryeol Ryu, Sang Ho Choi, Ju-Hoon Lee

**Affiliations:** Department of Food Science and Biotechnology, Institute of Life Science and Resources, Kyung Hee University, Yongin, Republic of Korea; National Research Laboratory of Molecular Microbiology and Toxicology, Department of Agricultural Biotechnology, and Center for Food Safety and Toxicology, Seoul National University, Seoul, Republic of Korea; Department of Food and Animal Biotechnology, Department of Agricultural Biotechnology, Research Institute of Agriculture and Life Sciences, and Center for Food and Bioconvergence, Seoul National University, Seoul, Republic of Korea; Department of Agricultural Biotechnology, Research Institute for Agriculture and Life Sciences, Seoul National University, Seoul, Republic of Korea

**Keywords:** Complete genome sequence, *Bacillus cereus*, Food-borne pathogen, Food poisoning, Diarrhea

## Abstract

Due to abundant contamination in various foods, the pathogenesis of *Bacillus cereus* has been widely studied in physiological and molecular level. *B. cereus* FORC_005 was isolated from a Korean side dish, soy sauce braised fish-cake with quail-egg in South Korea. While 21 complete genome sequences of *B. cereus* has been announced to date, this strain was completely sequenced, analyzed, and compared with other complete genome sequences of *B. cereus* to elucidate the distinct pathogenic features of a strain isolated in South Korea. The genomic DNA containing a circular chromosome consists of 5,349,617-bp with a GC content of 35.29 %. It was predicted to have 5170 open reading frames, 106 tRNA genes, and 42 rRNA genes. Among the predicted ORFs, 3892 ORFs were annotated to encode functional proteins (75.28 %) and 1278 ORFs were predicted to encode hypothetical proteins (748 conserved and 530 non-conserved hypothetical proteins). This genome information of *B. cereus* FORC_005 would extend our understanding of its pathogenesis in genomic level for efficient control of its contamination in foods and further food poisoning.

## Introduction

*Bacillus cereus* is one of the major food-borne pathogens, even though it is usually underreported due to its relatively mild symptoms and short duration [1, 2]. It has been known to contaminate diverse types of foods including meat, milk, eggs, and especially various vegetables. *B. cereus* also has been known to produce several pathogenic compounds and virulence factors including spores, dodecadepsipeptide cereulide, and enterotoxins. *B. cereus* has spore-forming ability so it may survive to cause problems in pasteurization and even sterilization in food processing, and this spore is highly hydrophobic to allow them to adhere to food transfer pipelines [1, 3, 4]. An extracellular protein, dodecadepsipeptide cereulide, is also known to be associated with emesis after food ingestion. In addition, *B. cereus* produces three different enterotoxins including hemolysin BL, nonhemolytic enterotoxin, and cytotoxin K, causing diarrhea after *B. cereus* infection [4, 5].

While the pathogenesis of *B. cereus* has been studied in physiological and molecular levels, characterization and pathogenesis studies of the genomes of *B. cereus* have been recently conducted to extend our understanding about its pathogenicity and virulence factors. To date, 21 different genomes of *B. cereus*, isolated from many other countries, have been completely sequenced and analyzed. However, the complete genome sequence of *B. cereus*, isolated from Korean foods, has never been announced previously. To elucidate the genome sequence and its genomic features of Korean *B. cereus*, its genome was completely sequenced, analyzed and compared with previously reported *B. cereus* complete genome sequences. Here, we present the complete genome sequence, annotation data, and genomic features of *B. cereus* FORC_005, which was isolated from a contaminated Korean side dish that caused food-borne illness in South Korea, and its evolutionary relationships with other previously reported complete genome sequences using comparative genomics.

## Organism information

### Classification and features

**Fig. 1:**
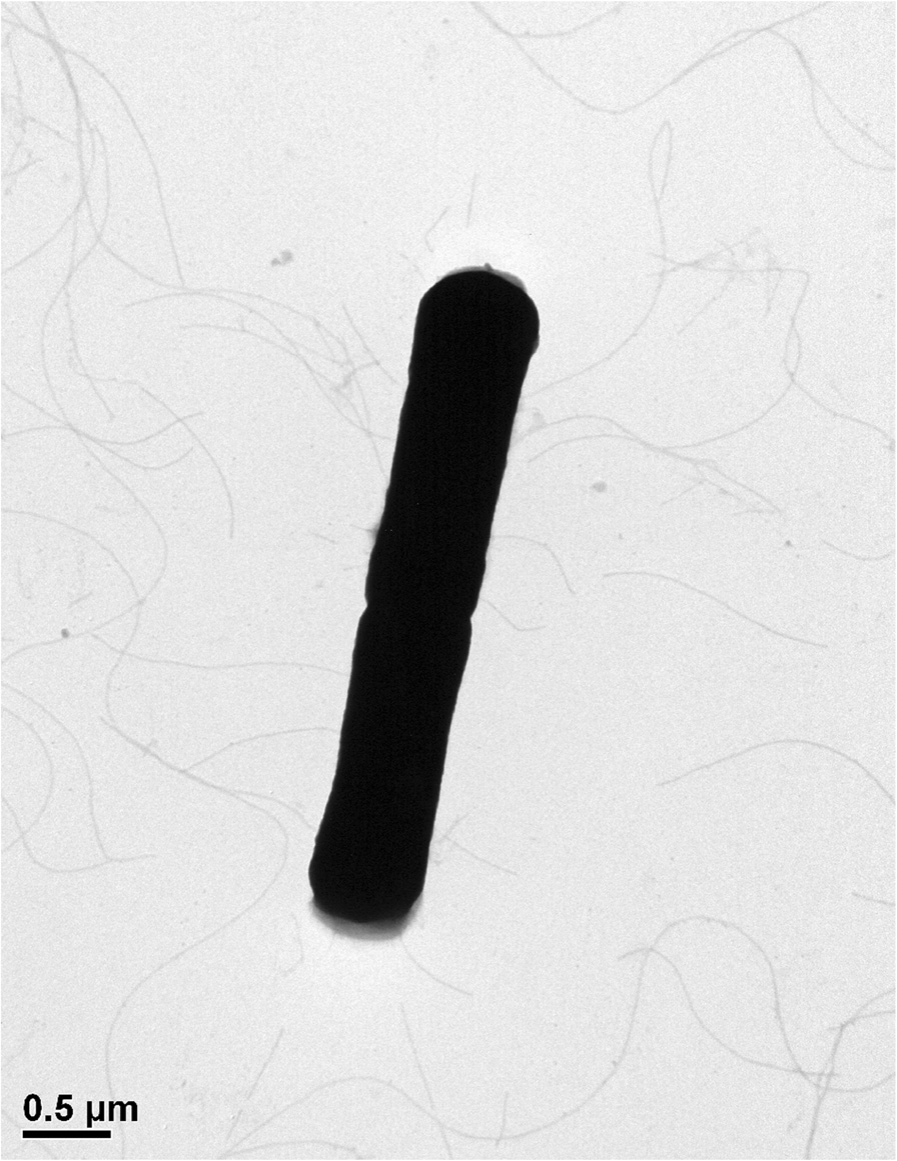
TEM image of the *B. cereus* FORC_005. The strain cells were negatively stained with 2 % (w/v) uranyl acetate and observed using TEM JEM-2100 (JEOL, Tokyo, Japan) at 200 kV

**Fig. 2:**
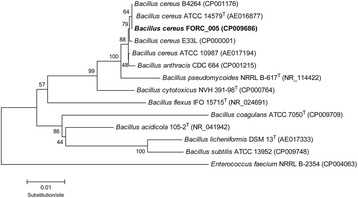
Phylogenetic tree highlighting the position of *B. cereus* strain FORC_005 relative to other species within the genus *Bacillus*. Alignment of 16S rRNA sequences was conducted using ClustalW [21], and the tree was generated using the Neighbor-joining algorithm with 1000 bootstraps, using MEGA 6 [22]. Type strains are labeled with superscript T, and the corresponding GenBank accession numbers are indicated in parentheses. *Enterococcus faecium* NRRL B-2354 was used as outgroup

*B. cereus* is a Gram-positive, rod-shaped, motile, and spore-forming bacterium. It is often found in various habitats including soil, water, and even food materials (fresh vegetables and food animals). In particular, this bacterium has been well-known food-borne pathogen causing diarrhea, vomiting, and nausea by enterotoxin production. It is a facultative anaerobe that can survive in the temperature range of 10–50 °C, pH range of 4.9–9.3, and salinity of up to 7.5 % NaCl. *B. cereus* belongs to the family *Bacillaceae*, the order Bacillales, the class Bacilli, and the phylum Firmicutes. In this study, *B. cereus* FORC_005 was isolated from a contaminated Korean side dish, soy sauce braised fish-cake with quail-egg, which was suspected to be an original pathogen of food-borne outbreak in March 2014, by Incheon Health and Environment Institute, South Korea. Morphology observation using Transmission Electron Microscopy (TEM; JEM–2100, JEOL, Tokyo, Japan) showed that the strain FORC_005 is rod-shaped with about 4 μm long and 0.5 μm wide in diameter, and can perform motility with peritrichous flagella, suggesting that the strain FORC_005 has typical morphology of *B. cereus* (Fig. 1 and Table 1). In addition, the 16S rRNA sequence analysis and phylogenetic tree analysis of the strain FORC_005 and other *Bacillus* species revealed that this strain was identified to *B. cereus* (data not shown) and positioned at the group of *B. cereus* in the phylogenetic tree, indicating that this strain indeed belongs to *B. cereus* (Fig. 2).

**Table 1:** Classification and general features of *Bacillus cereus* strain FORC_005 [6]

MIGS ID	Property	Term	Evidence code^a^
	Classification	Domain *Bacteria*	TAS [7]
		Phylum *Firmicutes*	TAS [8–10]
		Class *Firmibacteria*	TAS [8, 9, 11]
		Order *Bacillales*	TAS [8, 9, 12, 13]
		Family *Bacillaceae*	TAS [8, 9, 13, 14]
		Genus *Bacillus*	TAS [8, 9, 13, 15]
		Species *Bacillus cereus*	TAS [8, 9, 13, 16]
		Strain FORC_005	
	Gram stain	Positive	TAS [17, 18]
	Cell shape	Rod	TAS [17, 18]
	Motility	Motile with peritrichous flagella	TAS [17, 18]
	Sporulation	Endospore-forming	TAS [8]
	Temperature range	10 °C–50 °C	TAS [8, 17]
	Optimum temperature	28–35 °C	TAS [8]
	pH range; Optimum	4.9–9.3; 6.0–7.0	TAS [17]
	Carbon source	Glucose, Aesculin	TAS [19]
MIGS-6	Habitat	Ubiquitous; especially in soil	TAS [17, 18]
MIGS-6.3	Salinity	0–7.5 % NaCl (w/v)	TAS [17]
MIGS-22	Oxygen requirement	Facultative anaerobes	TAS [17, 18]
MIGS-15	Biotic relationship	free-living	NAS
MIGS-14	Pathogenicity	Diarrhea, emesis in human	NAS
MIGS-4	Geographic location	Incheon, South Korea	IDA
MIGS-23	Isolation	Korean soy sauce braised fish-cake with quail-egg	IDA
MIGS-5	Sample collection	March 2014	IDA
MIGS-4.1	Latitude	37.27 N	IDA
MIGS-4.2	Longitude	126.42 E	IDA
MIGS-4.4	Altitude	Not reported	

^a^ Evidence codes - *IDA* inferred from direct assay, *TAS* traceable author statement (i.e., a direct report exists in the literature), *NAS* non-traceable author statement (i.e., not directly observed for the living, isolated sample, but based on a generally accepted property for the species, or anecdotal evidence). These evidence codes are from the Gene Ontology project [20]

## Genome sequencing information

### Genome project history

The complete genome sequence and annotation data of *B. cereus* FORC_005 have been deposited in the GenBank database under the accession number CP009686. Genome sequencing of the strain FORC_005 is a part of the Food-borne Pathogen Omics Research Center project supported by Ministry of Food and Drug Safety South Korea, which aims for the collection and database construction of complete genome sequences of various food-borne pathogens in South Korea. A summary of the project information and its association with MIGS version 2.0 compliance [6] was presented in Table 2.

**Table 2:** Project information

MIGS ID	Property	Term
MIGS 31	Finishing quality	Finished
MIGS-28	Libraries used	Miseq 300 bp paired end, PacBio P4-C2 chemistry, size selected 10 kb library 2cell
MIGS 29	Sequencing platforms	Illumina MiSeq, PacBio RS II
MIGS 31.2	Fold coverage	494.66
MIGS 30	Assemblers	CLC genomics Workbench v7.0.4, PacBio SMRT Analysis v2.0
MIGS 32	Gene calling method	GeneMarkS, Glimmer3
	Locus Tag	FORC5
	Genbank ID	CP009686
	GenBank Date of Release	April 22, 2015
	GOLD ID	Gp0107273
	BIOPROJECT	PRJNA261557
MIGS 13	Source Material Identifier	FORC_005
	Project relevance	Agricultural

### Growth conditions and genomic DNA preparation

*B. cereus* FORC_005 was aerobically cultivated at 30 °C for 12 h with Brain Heart Infusion (BHI; Difco, Detroit, MI, USA) media, and the cells were harvested by centrifugation at 16,000 x g for 1 min. Total genomic DNA was extracted and purified using DNeasy Blood & Tissue Kit (Qiagen, Hilden, Germany), according to the manufacturer’s instructions for Gram-positive bacteria. Bacterial cells (about 2 X 10^9^ CFU) were harvested by centrifuging for 10 min at 5000 x g and pellet was resuspended with 180 μl of enzymatic lysis buffer (20 mM Tris · Cl, pH 8.0, 2 mM sodium EDTA, 1.2 % Triton X-100, and 20 mg/ml lysozyme) and this mixture was incubated at least 30 min at 37 °C. In addition to the mixture, 25 μl of proteinase K and 200 μl of Buffer AL, which are included in the kit, were mixed by vortexing before incubation at 56 °C for 30 min. And then 200 μl of absolute ethanol was added and mixed thoroughly by vortexing. The mixture was transformed to DNeasy Mini spin column in a new 2 ml collection tube and centrifuged at 6000 x g for 1 min to remove the flow-through. Then, 500 μl of Buffer AW2 was added and centrifuged for 3 min at 20,000 x g to wash the genomic DNA in the column. The column was placed into a clean 1.5 ml tube and 200 μl of Buffer AE was directly added onto the DNeasy membrane. After incubation at room temperature for 1 min, the column was centrifuged for 1 min at 6000 x g to elute the purified genomic DNA. The concentration and purity of the purified DNA was determined using a NanoVue spectrophotometer (GE Healthcare, Little Chalfont, UK).

### Genome sequencing and assembly

The genome sequence was determined using a hybrid-genome sequencing approach with PacBio RS II (Pacific Biosciences, Menlo Park, CA, USA) and Illumina MiSeq (Illumina, San Diego, CA, USA). Library construction for PacBio RS II was initialized by ligating universal hairpin adaptors to both ends of DNA fragments using SMRTbell Template Prep kit 1.0 (Pacific Biosciences), followed by purification using AMPure PB bead system (Pacific Biosciences) for the removal of small fragments sized <1.5 kb. Subsequent DNA polymerase binding with template DNAs was conducted using DNA/Polymerase Binding kit P6 v2 with C2 chemistry (Pacific Biosciences), followed by loading of SMRTbells using MagBeads kit (Pacific Biosciences) for greater number of reads at longer lengths per SMRT Cell. Sequencing was conducted using DNA sequencing Bundle 2.0 (Pacific Biosciences), on the PacBio RS II platform. 300 bp paired-end library for Illumina MiSeq (Illumina) was initialized by simultaneously fragmenting template DNAs and tagging them with sequencing adapters using Nextera DNA Sample Preparation kit and Index kit (Illumina), followed by purification of prepared template DNA fragments using MinElute reaction clean up kit (Illumina) and AMPure XP bead (Beckman Coulter, Brea, CA, USA). Sequencing was conducted using MiSeq Reagent kit (600 cycle; Illumina). All kits were used according to the manufacturer’s instructions. Sequencing reads from Illumina MiSeq system were assembled using the CLC Genomics Workbench v7.0.4 (CLC bio, Aarhus, Denmark), and the reads from PacBio system were assembled using the PacBio SMRT Analysis v2.0 (Pacific Biosciences). Finally, the initially assembled scaffolds were gathered and re-assembled to obtain one contig using CLC Genomics Workbench program.

### Genome annotation

**Fig. 3:**
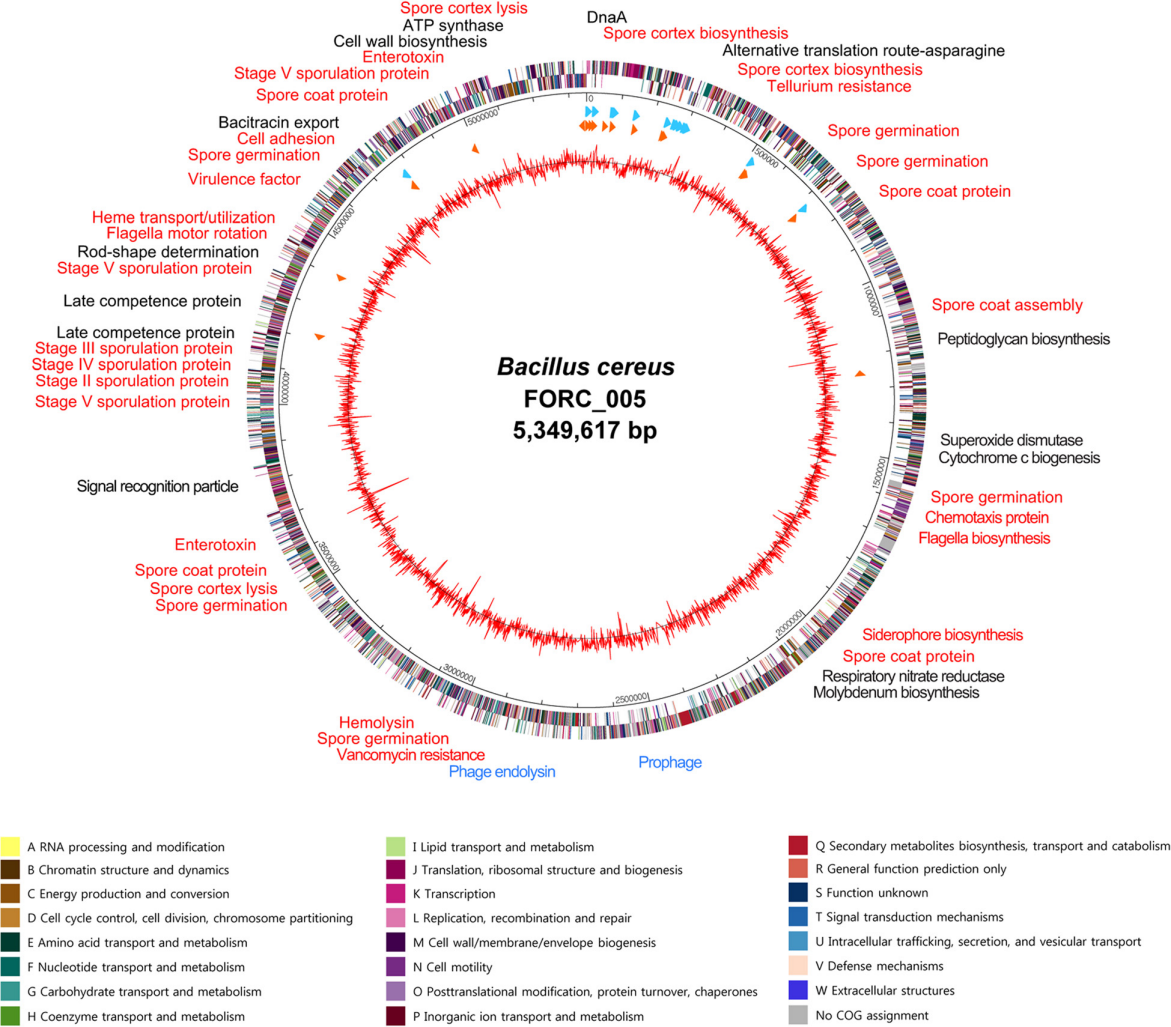
Genome map of *B. cereus* FORC_005. The outer circle indicates the location of all ORFs, and the inner circle with red peaks indicates the GC content. All ORFs were colored according to their COG functional groups. Sky-blue and orange arrows indicate rRNA, and tRNA genes, respectively. Gene clusters, virulence factors, and featural genes are labeled around the outer circle according to their approximate location. Labels were colored as follows; virulence-related genes in red, prophage-related genes in sky-blue, and other function-related genes in black

Initial prediction and annotation of all open reading frames, and tRNA/rRNA gene prediction was carried out using Glimmer3 by the Rapid Annotation using Subsystem Technology server [23], and was confirmed using the GeneMarkS ORF prediction program [24]. Predicted ribosome binding sites by RBSfinder (J. Craig Venter Institute, Rockville, MD, USA) were used to confirm the predicted ORFs. The Global Annotation of Multiplexed On-site Blasted DNA-Sequences program and InterProScan5 program with conserved protein domain databases were used for the annotation of confirmed ORFs [25, 26]. Artemis16 was used for handling of genome sequence and annotated data [27]. The functional categorization and classification of all predicted ORFs were conducted using the RAST server-based SEED viewer and Clusters of Orthologous Groups -based WebMGA programs [28, 29]. Circular genome map, showed in Fig. 3, was generated using GenVision (DNASTAR, Madison, WI, USA) based on all predicted ORFs with COG information, tRNAs and rRNAs, GC-content, and gene cluster information. Detection and identification of virulence factors were carried out using BLAST search with protein sequences of VFs in the database [30]. Signal peptides, transmembrane helices, and Clustered Regularly Interspaced Short Palindromic Repeats were identified by using SignalP server v.4.1 [31], TMHMM server v.2.0 [32], and CRISPRfinder [33], respectively.

## Genome properties

Table 3 contains the main FORC_005 statistics. It contains one chromosomal double-stranded DNA and no plasmid. The chromosome consists of 5,349,617 bp in DNA length with the GC content of 35.29 %, containing 5170 ORFs, 106 tRNA genes, and 42 rRNA genes consisting of 14 complete rRNA operons. Among the predicted 5170 ORFs, 3892 ORFs (75.28 %) were annotated to encode functional proteins and 1278 ORFs were hypothetical proteins (748 conserved and 530 non-conserved). In addition, 4202 ORFs (81.28 %) were assigned to the related COG functional categories, and are listed in Table 4.

**Table 3:** Genome statistics

Attribute	Value	% of Total^a^
Genome size (bp)	5,349,617	100.00
DNA coding (bp)	4,476,678	83.68
DNA G + C (bp)	1,887,736	35.29
DNA scaffolds	1	-
Total genes	5318	100.00
Protein coding genes	5170	97.22
RNA genes	148	2.78
Pseudo genes	134	2.52
Genes in internal clusters	0	0.00
Genes with function prediction	3892	73.19
Genes assigned to COGs	4202	81.28
Genes with Pfam domains	4718	88.72
Genes with signal peptides	383	7.20
Genes with transmembrane helices	1544	29.03
CRISPR repeats	4	-

^a^The total is based on either the size of the genome in base pairs or the total number of total gene in the annotated genome

**Table 4:** Number of genes associated with general COG functional categories

Code	Value	% age	Description
J	215	4.16	Translation, ribosomal structure and biogenesis
A	1	0.02	RNA processing and modification
K	378	7.31	Transcription
L	165	3.19	Replication, recombination and repair
B	1	0.02	Chromatin structure and dynamics
D	46	0.89	Cell cycle control, Cell division, chromosome partitioning
V	105	2.03	Defense mechanisms
T	230	4.45	Signal transduction mechanisms
M	249	4.82	Cell wall/membrane biogenesis
N	59	1.14	Cell motility
U	52	1.01	Intracellular trafficking and secretion
O	109	2.11	Posttranslational modification, protein turnover, chaperones
C	202	3.91	Energy production and conversion
G	264	5.11	Carbohydrate transport and metabolism
E	392	7.58	Amino acid transport and metabolism
F	121	2.34	Nucleotide transport and metabolism
H	171	3.31	Coenzyme transport and metabolism
I	127	2.46	Lipid transport and metabolism
P	229	4.43	Inorganic ion transport and metabolism
Q	92	1.78	Secondary metabolites biosynthesis, transport and catabolism
R	558	10.79	General function prediction only
S	436	8.43	Function unknown
-	968	18.72	Not in COGs

The total is based on the total number of protein coding genes in the genome

## Insights from the genome sequence

### Pathogenesis and virulence factors

Frequently, *B. cereus* causes diarrhea and emesis after ingestion of the contaminated food. These food-borne illnesses are reported to be associated with specific toxin genes. The genome analysis of *B. cereus* FORC_005 revealed that there are three major toxins including cytotoxin K, hemolysin BL, and non-hemolytic enterotoxin [5]. These toxins are involved in severe diarrhea after infection of *B. cereus*. Cytotoxin K is encoded by a single gene, *cytK* (FORC5_0979). However, other two toxins are encoded by two different gene clusters, a hemolysin BL gene cluster (*hblABDC*; FORC5_2954 to FORC5_2957) and a non-hemolytic enterotoxin gene cluster (*nheABC*; FORC5_1734 to FORC5_1736)). In addition, hemolysin III (*hlyIII*; FORC5_2063) was detected in the genome for additional hemolysis activity. Therefore, gene expression regulation of these toxin-associate genes may be key points for control and prevention of food poisoning after pathogenic *B. cereus* infection.

Anthrolysin O, one of the cholesterol-dependent cytolysins, was detected in the genome (FORC5_1940), which has been suggested to be a pore-forming protein often found in many Gram-positive bacteria. This hemolytic and cytolytic protein was reported to be associated with cholesterol binding in the host cell plasma membrane, pore formation via its oligomerization, and transfer of virulence factors to the host cell cytoplasm [34, 35]. In addition, an internalin (FORC5_1206) was detected in the genome, suggesting that it may play an important role in host cell invasion. The predicted functions of these two host invasive proteins in the genome revealed that regulation and control of this initial step in the occurrence of food-borne pathogenesis and illness may be important for the host protection.

*Bacillus* has been known to have two kinds of bacterial protection systems from the host cell immune system, including polysaccharide capsule (PSC) and polyglutamic acid capsule [36, 37]. While PSC biosynthesis is common in *B. cereus*, only PSC biosynthesis gene cluster (FORC5_4952 to FORC5_4971) was detected in the genome of *B. cereus* FORC_005, suggesting that the strain can protect itself from the host cell immune defense system for its further pathogenesis in the host cells. Therefore, this bacterial protection system is also considered as one of the virulence factors in *B. cereus*.

### Comparative genome analysis

**Fig. 4:**
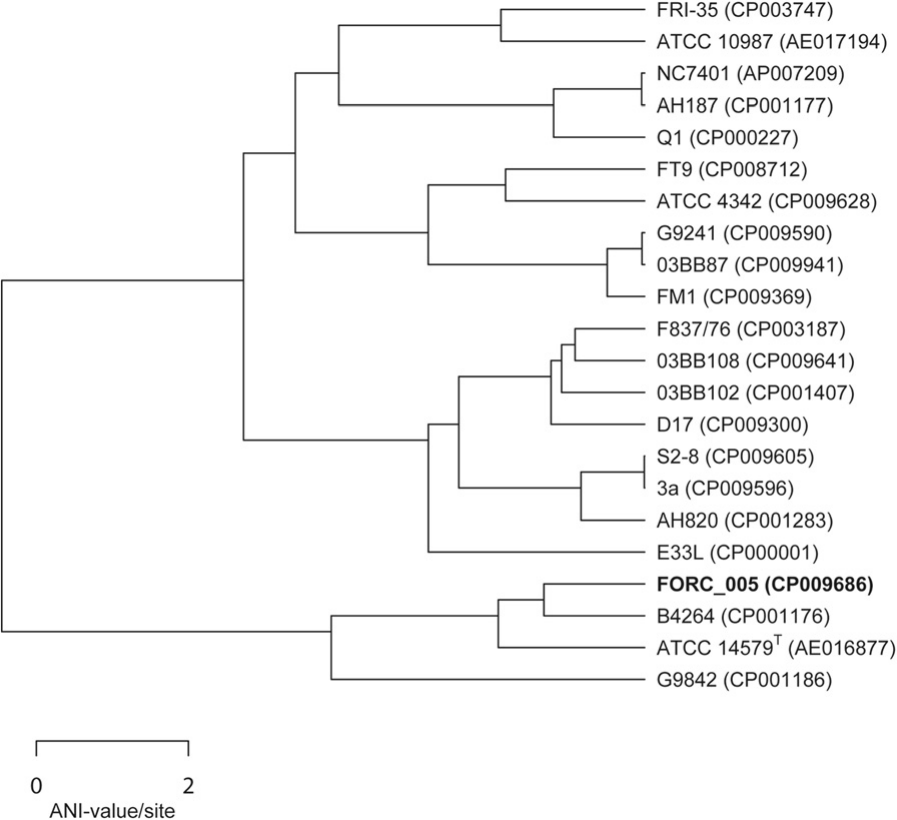
Genome tree highlighting the position of *B. cereus* FORC_005 relative to other *B. cereus* strains, based on the average nucleotide identity (ANI) value. ANI is a pairwise whole-genome comparing method for genetic relatedness between prokaryotic strains, and its values were calculated using JSpecies [39, 40], with nucleotide fragment length of 1020, based on the BLAST algorithm [41]. The genome tree was constructed using the R software according to the ANI values using the unweighted pair group method

To elucidate the evolutionary relationship of *B. cereus* FORC_005 with other complete genomes of *B. cereus*, 16S rRNA sequence-based phylogenetic tree analysis and whole genome-based average nucleotide identity (ANI) analysis were conducted. Comparative phylogenetic tree analysis revealed that *B. cereus* and *B. anthracis* strains formed a group including *B. cereus* FORC_005, suggesting that they may have been evolved from a common ancestor (Fig. 2). Subsequent ANI analysis using the complete genome sequences of strain FORC_005 and other 21 *B. cereus* strains revealed the closest evolutionary relationship between the strains FORC_005 and B4264 with the ANI value of 98.68 (Fig. 4). The strain B4264 was initially isolated from a male patient with a fatal pneumonia in 1969 [38], indicating that this strain is a clinical isolate. However, the strain FORC_005 was isolated from a *B. cereus**-*contaminated Korean food, suggesting that it may have pathogenesis for potential food-borne outbreak. Therefore, the genome information of the strain FORC_005 may be important to extend our knowledge about the study of food-borne outbreak via ingestion of the contaminated foods in genomic level, even though it is a food isolate *B. cereus* strain.

## Conclusions

While 21 complete genome sequences have been announced to date, all strains were originated from other countries. In this study, newly isolated *B. cereus* FORC_005 from a contaminated food in South Korea was selected for genomic study to elucidate the genomic pathogenesis of a *B. cereus* strain from South Korea and taxonomical location with complete genome sequences of other *B. cereus* strains.

The strain FORC_005 showed that anthrolysin O and internalin in the strain FORC_005 may occur or help the initial step of host cell invasion. In addition, the polysaccharide capsule biosynthesis gene cluster may protect the bacterial pathogen from the host cell immune defense system after host cell invasion. Subsequently, the pathogenesis-associated enterotoxin genes may cause severe diarrhea. The enterotoxin-associated genes in the genome encode three different enterotoxins including cytotoxin K, hemolysin BL, and non-hemolytic enterotoxin, suggesting that this strain may cause human diarrhea. Therefore, the genome of this strain has a complete set of genes or gene clusters for host cell invasion, bacterial protection from the host cell immune system, and enterotoxin production for diarrhea, suggesting that this strain is a food-borne pathogenic bacterium indeed. Comparative phylogenetic tree analysis and ANI analysis of the strain FORC_005 and other *B. cereus* strains revealed that a food isolate strain FORC_005 is the closest to a clinical isolate strain B4264, supporting this.

In conclusion, the genomic study of *B. cereus* FORC_005 provides important information about the genomic features and pathogenesis mechanism of a food isolate *B. cereus*, which is highly similar to a clinical isolate B4264. Furthermore, this genome information would be useful for development of novel biocontrol approach to regulate the pathogenesis of food isolate *B. cereus* strains.

## Abbreviations

FORC: Food-borne pathogen omics research Center
TEM: Transmission electron microscopy
PSC: Polysaccharide capsule
ANI: Average nucleotide identity
